# Supplementary material for: Photonic elementary cellular automata for simulation of complex phenomena
Source: Light Sci Appl. 2023 May 30;12:132. doi: 10.1038/s41377-023-01180-9 (PMC10229568; doi:10.1038/s41377-023-01180-9)
Supplement: Supplementary file 1 — Supplementary Information [file 41377_2023_1180_MOESM1_ESM.pdf]

Supplementary Information for Photonic Elementary Cellular Automata for Simulation of Complex Phenomena

Gordon H.Y. Li,[1] Christian R. Leefmans,[1] James Williams,[2] and Alireza Marandi[1,2]

[1] *Department of Applied Physics, California Institute of Technology, Pasadena, CA 91125, USA*

[2] *Department of Electrical Engineering, California Institute of Technology, Pasadena, CA 91125, USA*

1. EXPERIMENTAL SETUP

In Fig. S1, we present a detailed schematic of the experiment setup used to realize photonic elementary cellular automata (ECA). The setup may be divided into two segments: the optical segment and the electronic segment. The optical segment is essentially a multimode interferometer that takes a pulse train as its input and that outputs the interference between each pulse and its nearest neighbors. The electronic segment sets and stabilizes the relative phases interferometer arms, which helps us to implement different rules with the same hardware. It also converts the output of the interferometer to a binary signal and uses this binary signal to update the pulse train input into the interferometer.

The optical segment of our experimental setup begins at the mode-locked laser (MLL) shown in Fig. S1. The MLL outputs femtosecond optical pulses with a center wavelength of 1550 nm and a repetition period of $T_{\mathrm{R}} = 4$ ns. We stretch the pulses to ~ 5 ps with a 200 GHz Channel 34 filter to reduce the effects of dispersion.

After stretching the pulses, we tap 10% of the power with a 90:10 splitter and send it directly to a 600 MHz detector. We pass the RF output of the detector through a 300 MHz low pass filter, which isolates the 250 MHz component of the signal. This signal acts as a clock for our system's FPGA, which, as we shall explain shortly, generates the modulator driving signals for our experiment. Deriving the FPGA's clock directly from the optical pulse train eliminates any timing drift between our optical path and our electronic signals.

The 90% of the optical power that is not used to clock the FPGA is instead sent through two consecutive intensity modulators (IMs). The first IM, IM_{00}, converts the uniform input pulse train to a binary string that contains either an initial condition or the previous state of the ECA under study. The second IM, IM_{01}, helps us to achieve a better extinction ratio for the zeros in these binary strings.

After exiting the modulators, the binary pulse train passes through an erbium-doped fiber amplifer (EDFA) and another 200 GHz Channel 34 filter before finally reaching the interferometer. The pulses are first split between two paths at a 50:50 spliter. One of these paths leads to a second 50:50 splitter, where the pulses are again divided between another two paths. The paths after the second 50:50 splitter are labeled the $\pm 1T_{\mathrm{R}}$ delay lines. The lengths of these lines are chosen to delay advance the pulse train by one repetition period relative to the $0T_{\mathrm{R}}$ delay line, which is the other line after the first 50:50 splitter. The result

of delaying and advancing the pulse train in this manner is that we interfere each pulse in the $0T_{\mathrm{R}}$ delay line with its nearest-neighbor pulses once we recombine all of the paths.

The interferometer implements the rule for a particular ECA, so the output of the interferometer is the updated state of the ECA under study. To detect this state, we pass the output pulse train through another EDFA and 200 GHz Channel 34 filter. We then split the pulse train at a final 50:50 splitter and detect the signal on both a fast 5 GHz detector and a slow kHz detector. We send the RF output of the slow detector to the stabilization electronics for the interferometer arms, and we record the RF output of the 5 GHz detector on an oscilloscope. We then transfer the raw data trace from the oscilloscope to our lab computer.

We perform the thresholding operation electronically based on the peak voltage for each pulse time bin, which is necessary to prepare the next input to our ECA. After thresholding, we pass the binary values to the FPGA, which uses a digital-to-analog converter (DAC) to convert the array into an RF pulse pattern. This pulse pattern is amplified and sent to IM_{00} and IM_{01}, where it modulates the pulses from the MLL to produce the next input to the interferometer.

It is important to note that, while it was convenient for our proof-of-concept experiments, a digital computer is not necessary to perform the threshold function for our ECA. Indeed, one could imagine using a carefully calibrated comparator or another analog logic circuit to convert the output of our 5 GHz detector into a binary pulse train that can be fed back to the IMs before the interferometer.

The final components of our setup are the stabilization electronics. We utilize a Pound-Drever-Hall locking scheme [1] to lock the phases of the $\pm 1T_{\mathrm{R}}$ delay lines to the phase of the $0T_{\mathrm{R}}$ delay line. The RF output of our kHz detector is sent to two Red Pitaya STEMLabs, which contain built-in proportional-integral-derivative (PID) controllers. These devices output dither and control signals, which we combine and amplify with a custom printed circuit boards (PCBs). We send the outputs of these PCBs to fiber phase shifters in the $\pm 1T_{\mathrm{R}}$ delay lines to stabilize the phases of the lines.

A key benefit of using PDH locking for our photonic ECA experiments is that it enables us to independently lock each delay line in-phase or out-of-phase with the $0T_{\mathrm{R}}$ delay line. This ability allows us to either add or subtract the fields in the $\pm 1T_{\mathrm{R}}$ delay lines to or from the field in the $0T_{\mathrm{R}}$ delay line, and it is essential to enabling us to implement multiple ECA

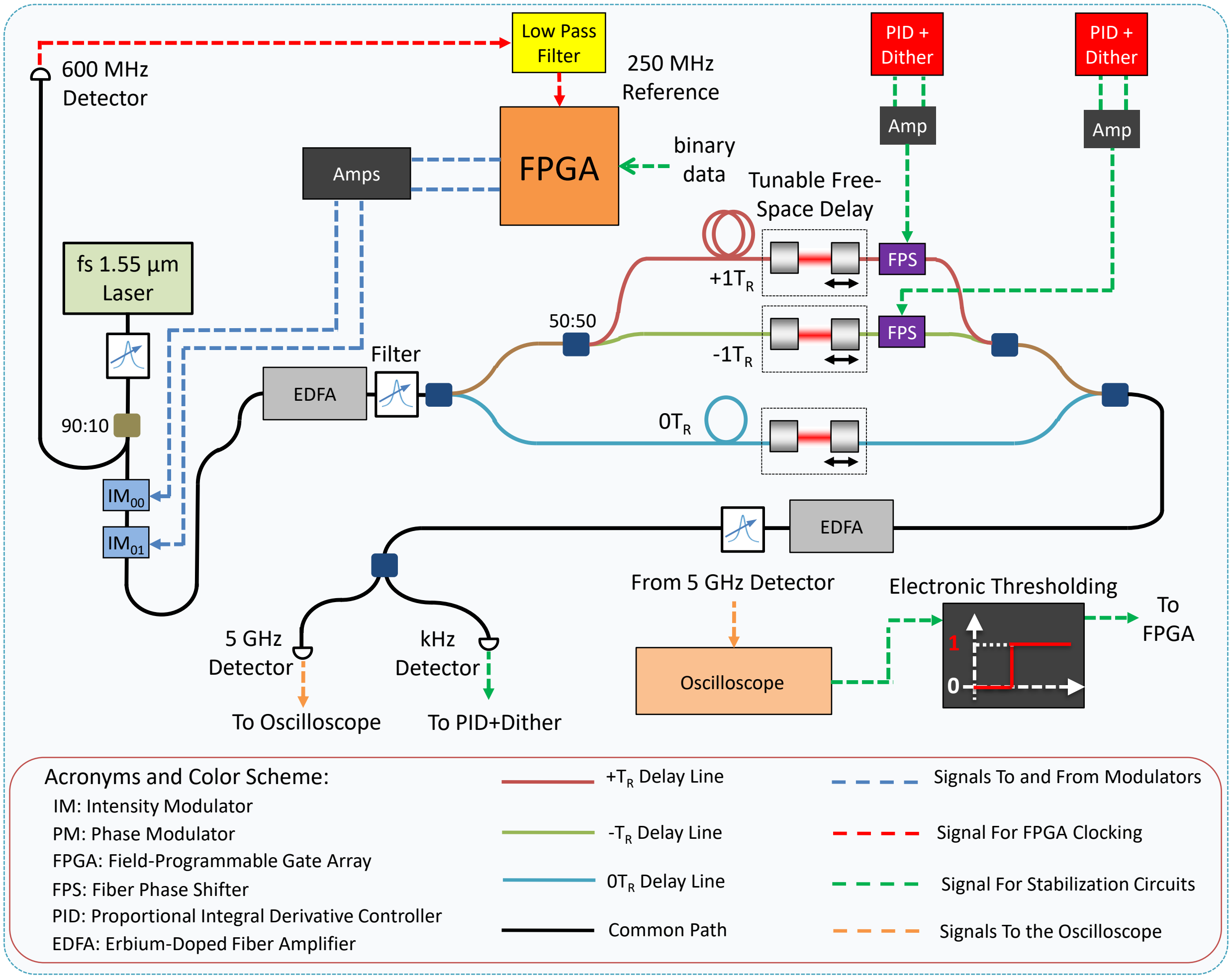

FIG. S1. **Detailed Schematic of Experimental Setup**

rules with the same photonic hardware.

2. EXPERIMENTAL PROCEDURE

We begin our experiments by configuring the lines of our interferometer to produce the correct rule for the ECA that we would like to study. Generating these rules involves adjusting both the relative intensities and phases between the three lines. We adjust the intensities in the lines by detuning the coupling in the free space delays shown in Fig. S1, and we set the relative phases to either 0 or π by changing the feedback signals from the PIDs used to stabilize the $\pm 1T_{\mathrm{R}}$ delay lines. In Supplementary Sec. 3, we discuss the particular mapping between each ECA studied in the main text and our photonic hardware.

After programming a particular rule into the interferometer, we program our FPGA to launch an initial condition into the system. We detect the response of the interferometer to this initial condition and average over five repetitions of this measurement. We then perform the threshold operation on the averaged data trace. As discussed above, the array of binary values that results from the threshold operation is the input to the next iteration of the ECA. We pass this array to our FPGA, which produces RF pulses to drive IM_{00} and IM_{01} in Fig. S1. In turn, these IMs to write the binary string onto the pulse train from our mode-locked laser, and this string corresponds to the updated state of our ECA. We repeat this process of performing the threshold operation on the averaged interferometer output and reinjecting the result of the threshold operation into the interferometer until we have iterated the ECA for the desired number of iterations.

To ensure that the phases of our delay lines are stable during our experiment, we track the output of our interferometer with a real-time simulation of the ECA rule. If the binary array produced by our threshold operation differs from what we would expect in our simulation - even by just a single cell - we rerun that iteration of the ECA until the iteration succeeds. Without this form of error-correction, the system would behave as stochastic ECA. In practice, the number of failed iterations depends on the particular ECA rule being implemented, but the iteration success rate is high for each of the experiments presented in the main text. The success rates are $\sim 100\%$ for Rule 90, $> 81\%$ for Rule 30. . . , and $> 90\%$ for Rule 54. The iteration success rates for Rule 30 and Rule 54 appear lower than ideal because of a lower interference visibility compared to Rule 90. Note that the individual cell-wise bit error rate is $< 10^{-3}$ for each of the rules, indicating good cell-wise fidelity. The failures that do occur are due to instability in the delay line phases; power drift in the input pulse train, which can causes pulses that are close to one side of the threshold to cross to the other side; or insufficient averaging over the fluctuations in the recorded traces, which can cause some pulses to fluctuate across the threshold. While this last cause can be alleviated by averaging over additional traces, there is a trade-off between the likelihood that an iteration will fail and the time it takes to run our experiments. The longer we run our experiment, the more susceptible it is to fail because of drift in the input power.

3. MAPPING ECA RULES TO PHOTONIC HARDWARE

Here, we provide an explicit mapping between each of the ECA rules studied in the main text and the paramters of our photonic platform. Setting the desired ECA rule involves adjusting both the relative intensities and phases between the three delay lines. We adjust the intensities in the lines by detuning the coupling in the free space delays shown in Supplementary Information Fig. 1, and we set the relative phases to either 0 or π by changing the feedback signals from the PIDs used to stabilize the $\pm 1T_{\mathrm{R}}$ delay lines. A relative phase of 0 produces constructive interference between two delay lines, and conversely a relative phase of π produces destructive interference. Therefore, the result of the $\pm 1T_R$ delay lines, tuning the VOAs, and setting relative phases can be summarized as:

$$y_i(t) = a_{-1}x_{i-1}(t) + a_0 x_i(t) + a_1 x_{i+1}(t) \ , \tag{1}$$

where $x_i(t) \in \mathbb{Z}_2$ is the amplitude of the ith light pulse in the tth iteration before being split into the delay lines, $y_i(t)$ is the amplitude of the light pulse after recombining delay lines, and $\{a_{-1}, a_0, a_1\} \in [-1, 1]$ represent the relative strengths and phases of the different delay lines. The light pulse amplitude $y_i(t)$ is converted to an intensity $|y_i(t)|^2$ after passing through the photodetector and then optoelectronic thresholding performs the function:

$$x_i(t+1) = H\left(|y_i(t)|^2 - b\right) \ , \tag{2}$$

where $H(x)$ is the Heaviside step function, $b \in \mathbb{R}$ is the thresholding value, and $x_i(t+1)$ is the output result to be reinjected as the light pulse amplitude in the next iteration. As a result of thresholding, any light intensity $|y_i(t)|^2 < b$ represents a dead cell, and conversely any light intensity $|y_i(t)|^2 > b$ represents a live cell. Examples of possible mappings for each ECA rule are listed below. Note that the set of parameters $\{a_{-1}, a_0, a_1, b\}$ implementing a particular rule in our photonic system are not unique, many different sets of parameters can yield the same rule.

Rule 90

The truth table for ECA Rule 90 is shown in the main text Fig. 1(b). It's equivalent Boolean form is:

$$x_i(t+1) = x_{i-1}(t) \oplus x_{i+1}(t) \ . \tag{3}$$

The table below shows how this rule can be reconstructed using the linear interference, photodetection and thresholding in our photonic system.

x_{i-1}	x_i	x_{i+1}	$y_i = -1x_{i-1} + 0x_i + 1x_{i+1}$	$\lvert y_i\rvert^2$	$H(\lvert y_i\rvert^2 - 0.5)$	$\mathbf{x_{i-1} \oplus x_{i+1}}$
0	0	0	0	0	0	**0**
0	0	1	1	1	1	**1**
0	1	0	0	0	0	**0**
0	1	1	1	1	1	**1**
1	0	0	-1	1	1	**1**
1	0	1	0	0	0	**0**
1	1	0	-1	1	1	**1**
1	1	1	0	0	0	**0**

TABLE I. Truth table and explicit mapping of ECA Rule 90 to photonic hardware.

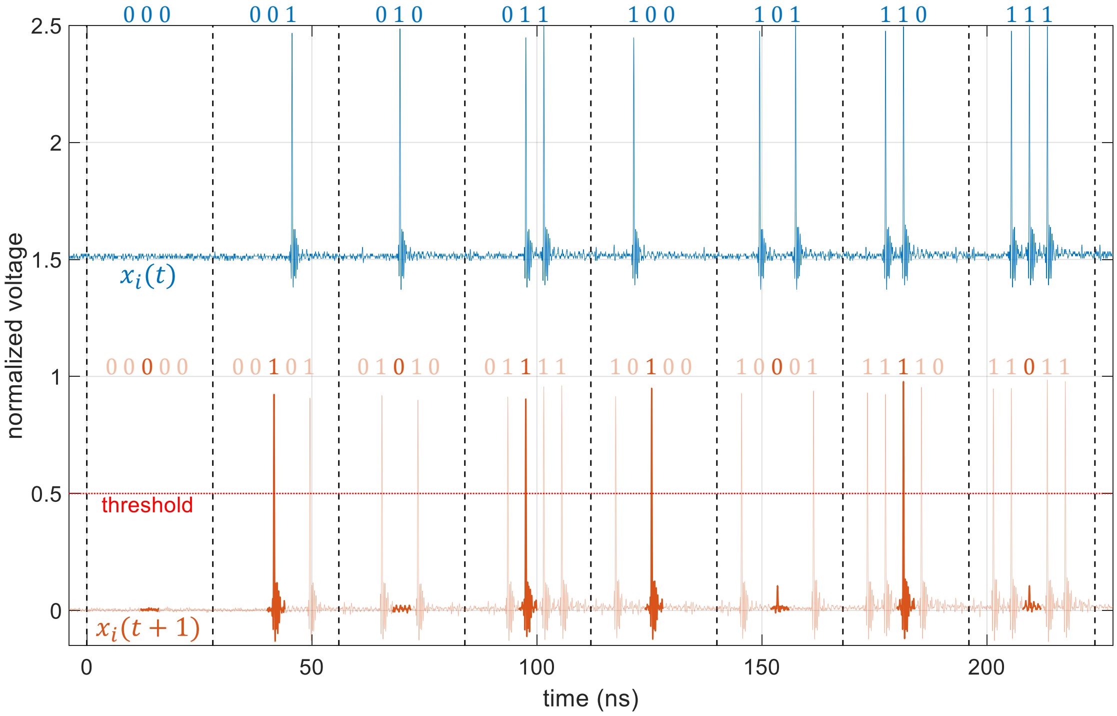

FIG. S2. **Experimentally computing the truth table for ECA Rule 90.**

Fig. S2 shows an experimentally obtained time trace for a single iteration that explicitly computes the truth table for ECA Rule 90 given the 8 possible initial conditions for a 3-cell

neighborhood. The initial condition $x_i(t)$ is shown is blue (vertically separated for easier viewing), and the resultant cell state $x_i(t+1)$ is shown below in red. The voltage represents the detected optoelectronic signal $|y_i|^2$. The dotted red line indicates the electronic thresholding value that will binarize the peak voltages in each time bin, which shows that the output value $x_i(t+1) = x_{i-1}(t) \oplus x_{i+1}(t)$ is implemented as intended.

Rule 30

The truth table for ECA Rule 30 is shown in the main text Fig. 3(a). It's equivalent Boolean form is:

$$x_i(t+1) = x_{i-1}(t) \oplus (x_i(t) \lor x_{i+1}(t)) \ . \tag{4}$$

The table below shows how this rule can be reconstructed using the linear interference, photodetection and thresholding in our photonic system.

x_{i-1}	x_i	x_{i+1}	$y_i = 1x_{i-1} - 0.66x_i - 0.66x_{i+1}$	$\lvert y_i\rvert^2$	$H(\lvert y_i\rvert^2 - 0.4)$	$\mathbf{x_{i-1} \oplus (x_i \lor x_{i+1})}$
0	0	0	0	0	0	**0**
0	0	1	-0.66	~ 0.44	1	**1**
0	1	0	-0.66	~ 0.44	1	**1**
0	1	1	-1.32	~ 1.74	1	**1**
1	0	0	1	1	1	**1**
1	0	1	0.34	~ 0.12	0	**0**
1	1	0	0.34	~ 0.12	0	**0**
1	1	1	-0.32	~ 0.10	0	**0**

TABLE II. Truth table and explicit mapping of ECA Rule 30 to photonic hardware.

Rule 54

The truth table for ECA Rule 54 is shown in the main text Fig. 4(a). It's equivalent Boolean form is:

$$x_i(t+1) = (x_{i-1}(t) \lor x_{i+1}(t)) \oplus x_i(t) \ . \tag{5}$$

The table below shows how this rule can be reconstructed using the linear interference, photodetection and thresholding in our photonic system.

x_{i-1}	x_i	x_{i+1}	$y_i = -0.66x_{i-1} + 1x_i - 0.66x_{i+1}$	$\|y_i\|^2$	$H(\|y_i\|^2 - 0.4)$	$\mathbf{x_{i-1} \oplus (x_i \vee x_{i+1})}$
0	0	0	0	0	0	**0**
0	0	1	-0.66	~ 0.44	1	**1**
0	1	0	1	1	1	**1**
0	1	1	0.34	~ 0.12	0	**0**
1	0	0	-0.66	~ 0.44	1	**1**
1	0	1	-1.32	~ 1.74	1	**1**
1	1	0	0.34	~ 0.12	0	**0**
1	1	1	-0.32	~ 0.10	0	**0**

TABLE III. Truth table and explicit mapping of ECA Rule 54 to photonic hardware.

We focused only on 3 ECA rules that exhibited some representative complex phenomena, however, this kind of photonic computer can be used to implement all 256 possible ECA rules. To show this more clearly, we give examples of explicit photonic hardware mappings for all 256 ECA rules in Table. IV. Note that the photonic hardware mappings are not unique, there exist many possible mappings for each rule. Only the 88 inequivalent ECA rules are listed since some rules are equivalent up to reflection or complementing (equivalent rules in parenthesis). We slightly modify Eq. 1 to allow for the possible addition of a constant bias term $d \in [-1, 1]$, which corresponds to an extra interference path with a constant input:

$$y_i(t) = a_{-1}x_{i-1}(t) + a_0x_i(t) + a_1x_{i+1}(t) + d \ , \tag{6}$$

and introduce an upper thresholding value $c \in \mathbb{R}$ such that:

$$x_i(t+1) = \begin{cases} 1, & \text{if } b \leq |y_i(t)|^2 \leq c \\ 0, & \text{otherwise} \end{cases} \ . \tag{7}$$

The Heaviside step function $H(x)$ used in Eq. 2 is just a special case of Eq. 7 with $c = \infty$. An upper threshold is needed to implement odd-numbered ECA rules, which will have update rules satisfying $f(0, 0, 0) = 1$. Alternatively, a single threshold value can be used like in Eq. 2 if the linear weights are allowed to be complex, $a_i \in \mathbb{C}$, by controlling the full complex amplitude of light.

Rule(s)	a_{-1}	a_0	a_1	b	c	d
0 (255)	0	0	0	0	0	0
1 (127)	1	1	1	0	0.9	0
2 (16, 191, 247)	1	1	0.9	0.1	0.9	0
3 (17, 63, 119)	1	1	0.9	0	0.9	0
4 (223)	1	0.9	1	0.1	0.9	0
5 (95)	1	0.9	1	0	0.9	0
6 (20, 159, 215)	1	0.9	0.9	0.1	0.9	0
7 (21, 31, 87)	1	0.9	0.9	0	0.9	0
8 (64, 239, 253)	1	0.9	0.9	1.1	3.6	0
9 (65, 111, 125)	1	0.9	-0.5	0	0.2	0
10 (80, 175, 245)	1	0.9	-0.4	0.1	0.3	0
11 (47, 81, 117)	1	0.9	-0.1	0	0.8	0
12 (68, 207, 221)	1	0.9	0	0.1	0.9	0
13 (69, 79, 93)	1	0.4	-0.5	0	0.2	0
14 (84, 143, 213)	1	0.5	0.4	0.1	0.9	0
15 (85)	1	0.9	0	0	0.9	0
18 (183)	1	0.9	1	0.9	3.6	0
19 (55)	0.9	1	0.9	0	0.9	0
22 (151)	1	1	1	0.1	3.9	0
23	1	1	1	0	3.9	0
24 (66, 189, 231)	1	0.9	0.9	0.9	3.6	0
25 (61, 67, 103)	0.4	1	-0.9	0	0.2	0
26 (82, 167, 181)	1	0.8	0.9	0.7	3.2	0
27 (39, 53, 83)	1	0.8	0.9	0.1	1.6	-0.5
28 (70, 157, 199)	1	0.9	0.8	0.7	3.2	0
29 (71)	1	0.9	0.8	0.1	1.6	-0.5
30 (86, 135, 149)	1	0.9	0.9	0.1	3.6	0
32 (251)	1	0.9	1	3.7	5	0
33 (123)	1	0.6	-1	0	0.1	0
34 (48, 187, 243)	1	0.9	-0.5	0.2	0.8	0
35 (49, 59, 115)	0.9	1	-0.1	0	0.8	0
36 (219)	1	0.9	-0.1	0.7	0.9	0

Continued on next page...

Rule(s)	a_{-1}	a_0	a_1	b	c	d
37 (91)	1	0.4	-0.9	0	0.2	0
38 (52, 155, 211)	1	0.9	-0.5	0.2	0.9	0
40 (96, 235, 249)	1	1	0.9	1.1	3.9	0
41 (97, 107, 121)	1	1	-0.6	0	0.3	0
42 (112, 171, 241)	1	1	-0.4	0.1	0.9	0
43 (113)	1	1	-0.1	0	0.9	0
44 (100, 203, 217)	1	0.9	-0.1	0.1	0.9	0
45 (75, 89, 101)	1	0.8	-0.9	0	0.8	0
46 (116, 139, 209)	1	0.9	-0.4	0.1	0.9	0
50 (179)	0.5	1	0.4	0.1	0.9	0
51	0.9	1	0	0	0.9	0
54 (147)	1	0.9	-0.5	0.2	1.9	0
56 (98, 185, 227)	1	0.9	0.8	0.9	3.6	0
57 (99)	0.8	1	-0.9	0	0.8	0
58 (114, 163, 177)	0.9	1	-0.4	0.1	0.9	0
60 (102, 153, 195)	1	1	0.9	0.9	3.9	0
62 (118, 131, 145)	1	1	0.9	0.1	3.9	0
72 (237)	1	0.9	1	1.1	3.9	0
73 (109)	1	-0.6	1	0	0.3	0
74 (88, 173, 229)	1	-0.1	0.9	0.1	0.9	0
76 (205)	1	-0.4	1	0.1	0.9	0
77	1	-0.1	1	0	0.9	0
78 (92, 141, 197)	1	-0.4	0.9	0.1	0.9	0
90 (165)	1	0.9	1	0.9	3.9	0
94 (133)	1	0.9	1	0.1	3.9	0
104 (233)	1	1	1	1.1	5	0
105	1	1	1	0.1	5	-0.7
106 (120, 169, 225)	1	-0.1	-0.5	0.2	0.9	0
108 (201)	1	-0.4	-0.3	0.1	0.9	0
110 (124, 137, 193)	1	-0.4	-0.4	0.1	0.9	0
122 (161)	1	0.9	1	0.9	5	0
126 (129)	1	1	1	0.1	5	0

Continued on next page...

Rule(s)	a_{-1}	a_0	a_1	b	c	d
128 (254)	1	1	0.2	4.1	5	0
130 (144, 190, 246)	1	0.8	-0.9	0.7	0.9	0
132 (222)	1	0.9	-1	0.1	0.9	0
134 (148, 158, 214)	1	0.8	-0.9	0.1	0.9	0
136 (192, 238, 252)	1	0.4	-0.9	0.2	0.8	0
138 (174, 208, 244)	1	0.4	-0.9	0.2	0.9	0
140 (196, 206, 220)	1	0.6	-1	0.1	0.9	0
142 (212)	1	0.5	-0.9	0.1	0.9	0
146 (182)	1	0.8	-0.9	0.7	3.2	0
150	1	1	-0.6	0.2	3.9	0
152 (188, 194, 230)	1	0.8	-0.7	0.1	0.3	-0.6
154 (166, 180, 210)	1	0.4	-0.9	0.2	1.9	0
156 (198)	1	-0.6	0.1	0.2	1.2	0
160 (250)	1	0.5	0.7	2.3	5	0
162 (176, 186, 242)	1	0.4	-0.5	0.2	0.9	0
164 (218)	1	0.8	-0.9	0	0.2	-0.5
168 (224, 234, 248)	1	0.1	-0.6	0.1	0.3	0
170 (240)	1	0.2	-0.6	0.1	0.9	0
172 (202, 216, 228)	1	0.1	-0.9	0.1	0.3	0.3
178	1	0.4	-0.5	0.2	1.9	0
184 (226)	1	0.9	-0.8	0.1	0.3	-0.6
200 (236)	1	-0.3	-0.1	0.1	0.8	0
204	1	-0.4	0	0.1	0.9	0
232	1	1	0.2	1.1	5	0

TABLE IV: Example mappings of all 256 possible ECA rules (equivalent rules are listed in parenthesis if they exist).

4. SCALING TO TERAHERTZ CLOCK RATES

The pulse repetition rate can be increased to achieve potentially terahertz clock rates by using a similar time-multiplexing delay-line technique as in the current experiments. However, instead of interacting neighbouring pulses in the ECA, we can interleave pulses to

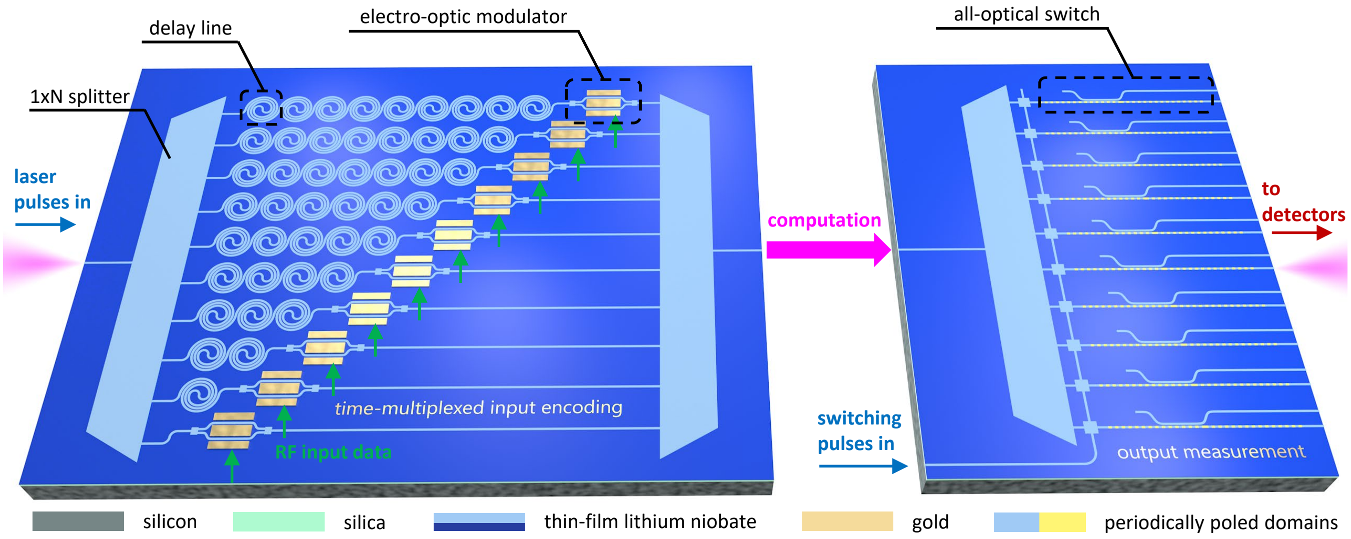

FIG. S3. Time-multiplexing method for achieving terahertz pulse repetition rates.

effectively increase the repetition rate. For example, suppose we want to achieve a 1 THz pulse repetition rate. Then, we can utilize a setup as shown in Fig. 3. In the left part of the figure, we illustrate time-multiplexed input encoding for the initial condition. Consider a 100 GHz repetition rate femtosecond laser source, but integrated electro-optic modulators (EOMs) that only have 100 GHz bandwidth [2]. We can split the input pulse train into $N = 10$ delay lines, each offset by a relative delay of 10 ps and then individually modulate each of these delay lines with a 100 GHz EOM. Then, upon recombination, we have effectively produced a pulse train with a 1 THz repetition rate. This initial condition can then be used as the input to our proposed all-optical ECA in which the nonlinear waveguides for all-optical thresholding have optical bandwidth > 10 THz. On the other hand, electronic thresholding and feedback is limited to the electronic bandwidth of < 10 GHz, which cannot support the ultrafast repetition rates attainable by all-optical methods. In the right part of the figure, we present a scheme to measure this 1 THz repetition rate pulse train for outputs by selectively switching the pulse train into 10 sets of 100 GHz repetition rate pulse trains using a set of 10 all-optical switches [3]. These 100 GHz repetition rate channels are then amenable to direct photodetection. Therefore, we see that the repetition rate of the laser source is not the limiting factor since we can always employ time-multiplexing methods to achieve any desired pulse repetition rate.

5. RULE 54 SPACE-TIME DIAGRAMS WITHOUT FILTERING

We applied tiling theory to filter out the periodic background, i.e. the *ether*, from the space-time diagrams of ECA Rule 54 shown in the main text Fig. 4. For completeness, we also show the same space-time diagrams below but without filtering.

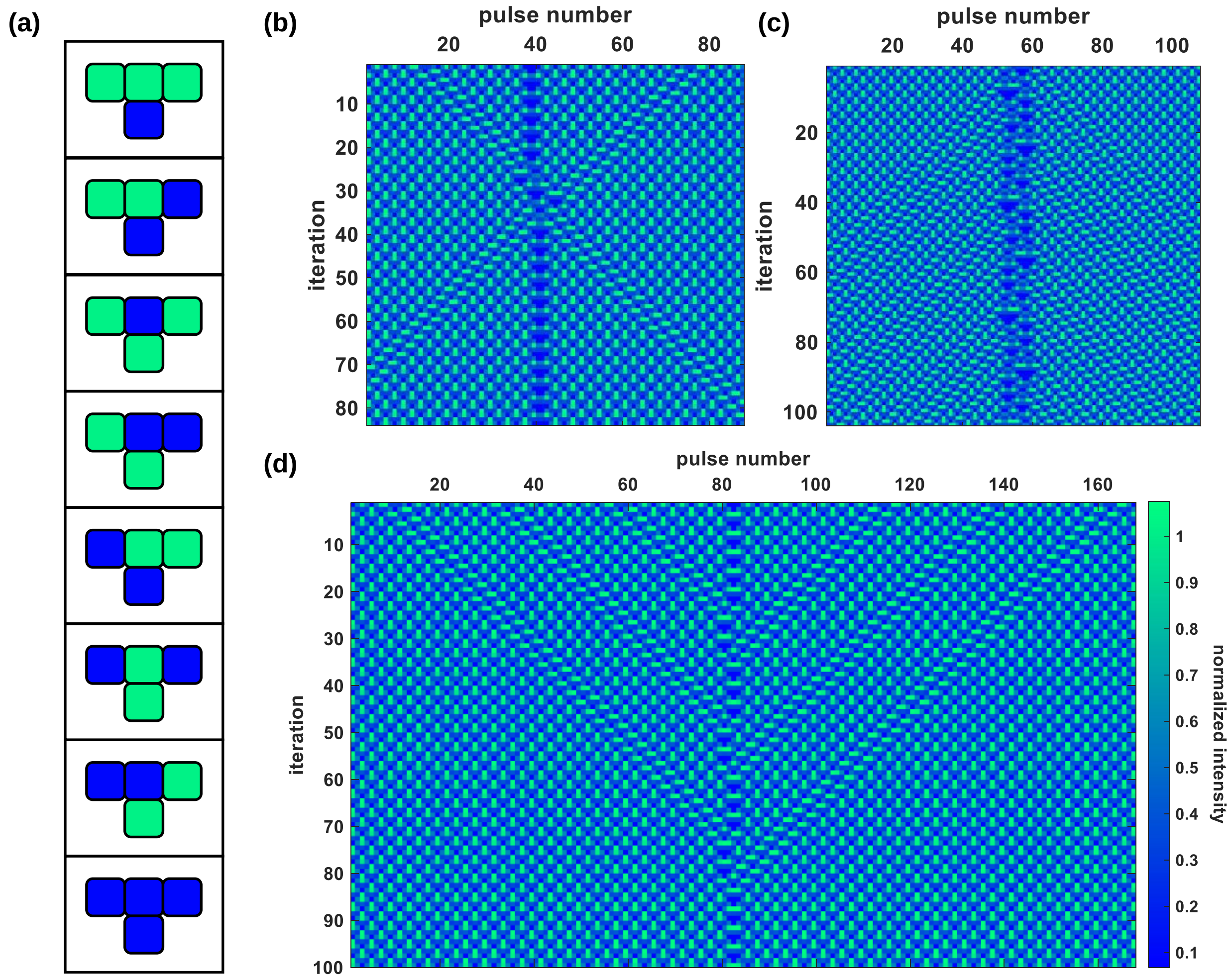

FIG. S4. **Soliton-like and glider interactions produced by ECA Rule 54 in the photonic hardware.** (a) Truth table showing the update for ECA Rule 54. Space-time diagrams of ECA Rule 54 *without* periodic background filtered out, showing (b) glider collision, (c) glider gun, and (d) black hole.

[1] E. D. Black, An introduction to Pound–Drever–Hall laser frequency stabilization, American Journal of Physics **69**, 79 (2001).

[2] C. Wang, M. Zhang, X. Chen, M. Bertrand, A. Shams-Ansari, S. Chandrasekhar, P. Winzer, and M. Lončar, Integrated lithium niobate electro-optic modulators operating at cmos-compatible voltages, Nature **562**, 101 (2018).

[3] Q. Guo, R. Sekine, L. Ledezma, R. Nehra, D. J. Dean, A. Roy, R. M. Gray, S. Jahani, and A. Marandi, Femtojoule femtosecond all-optical switching in lithium niobate nanophotonics, Nature Photonics **16**, 625 (2022).
